# Dietary Protein Consumption and the Risk of Type 2 Diabetes: A Systematic Review and Meta-Analysis of Cohort Studies

**DOI:** 10.3390/nu9090982

**Published:** 2017-09-06

**Authors:** Shuang Tian, Qian Xu, Ruyue Jiang, Tianshu Han, Changhao Sun, Lixin Na

**Affiliations:** 1 Nutrition Department, Longgang Hospital of Traditional Chinese Medicine, Shenzhen 518000, Guangdong, China; 18504541023@163.com; 2 National Discipline, Department of Nutrition and Food Hygiene, School of Public Health Harbin Medical University, Harbin 150081, China; XUQIAN9188@163.com (Q.X.); jruyue@163.com (R.J.); snowcalendar@126.com (T.H.)

**Keywords:** protein, diabetes, systematic review, meta-analysis

## Abstract

Recently, some studies have focused on the relationship between dietary protein intake and the risk of type 2 diabetes mellitus (T2DM), but the conclusions have been inconsistent. Therefore, in this paper, a systematic review and meta-analysis of cohort studies regarding protein consumption and T2DM risk are conducted in order to present the association between them. We searched the PubMed and Embase databases for cohort studies on dietary protein, high-protein food consumption and risk of T2DM, up to July 2017. A summary of relative risks was compiled by the fixed-effect model or random-effect model. Eleven cohort studies regarded protein intake and T2DM (52,637 cases among 483,174 participants). The summary RR and 95% CI (Confidence Interval) of T2DM was 1.12 (1.08–1.17) in all subjects, 1.13 (1.04–1.24) in men, and 1.09 (1.04–1.15) in women for total protein; 1.14 (1.09–1.19) in all subjects, 1.23 (1.09–1.38) in men, and 1.11 (1.03–1.19) in women for animal protein; 0.96 (0.88–1.06) in all subjects, 0.98 (0.72–1.34) in men, and 0.92 (0.86–0.98) in women for plant protein. We also compared the association between different food sources of protein and the risk of T2DM. The summary RR (Relative Risk) and 95% CI of T2DM was 1.22 (1.09–1.36) for red meat, 1.39 (1.29–1.49) for processed meat, 1.03 (0.89–1.17) for fish, 1.03 (0.64–1.67) for egg, 0.89 (0.84–0.94) for total dairy products, 0.87 (0.78–0.96) for whole milk, 0.83 (0.70–0.98) for yogurt, 0.74 (0.59–0.93) in women for soy. This meta-analysis shows that total protein and animal protein could increase the risk of T2DM in both males and females, and plant protein decreases the risk of T2DM in females. The association between high-protein food types and T2DM are also different. Red meat and processed meat are risk factors of T2DM, and soy, dairy and dairy products are the protective factors of T2DM. Egg and fish intake are not associated with a decreased risk of T2DM. This research indicates the type of dietary protein and food sources of protein that should be considered for the prevention of diabetes.

## 1. Introduction

Type 2 diabetes is rapidly increasing in the world. Ninety percent of patients with diabetes have type 2 diabetes [1]. Type 2 diabetes patients are at increased risk of cardiovascular disease, neuropathies, nephropathies, gangrene, and leg ulcers [2]. From the date of diagnosis, patients with T2DM have to face at least eight years of economic burden [3].

Evidence suggests that dietary factors may influence the risk of T2DM [4]. Many previous studies have focused on dietary macronutrient intake associated with diabetes risk [5,6], but the main content of those studies was carbohydrates and fats. Recently, some studies focused on the relationship between dietary protein intake and the risk of T2DM. High-protein diets have shown beneficial effects on glucose homeostasis in some short–term trials [7,8]. Subsequent longitudinal studies have evaluated the associations between dietary protein intake and risk of T2DM as well as the type of dietary protein and risk of T2DM.A study focusing on the Mediterranean islands showed that animal protein consumption was associated with a higher prevalence of diabetes among the elderly, and a recommended range of protein from plant sources appears was seen to be considerably protective [9]. Some other publications have also reported an increased risk of T2DM with a high intake of total protein [10,11,12,13,14] and animal protein [10,13,14,15], and a decreased risk of T2DM with a high intake of plant protein [14,15]. However, there have been no reports that how an association between T2DM and total protein [15,16,17,18], animal protein [16,17,18] and plant protein intake or a high risk of T2DM with a high intake of plant protein [10,13].The association between dietary protein and T2DM is still debated.

In the present study, we conducted a systematic review and meta-analysis of cohort studies to clarify the association of protein consumption with risk of T2DM. In order to provide a better dietary instruction for the lay public, we also conducted a systematic review and meta-analysis of cohort studies to study the association between different kinds of high-protein food and the risk of T2DM.

## 2. Materials and Methods

### 2.1. Search Strategy

We searched relevant studies from the Embase and PubMed electronic databases from their starting dates to July 2017. 

Protein: search items included: ‘dietary protein’ or ‘protein intake’ or ‘plant protein’ or ‘animal protein’ or ‘food’ and ‘diabetes’ or ‘diabetes mellitus’ or ‘T2DM’ and ‘population’ or ‘human’.

Meat: search items included: ‘red meat’ or ‘processed meat’ or ‘food’ and ‘diabetes’ or ‘diabetes mellitus’ or ‘T2DM’ and ‘population’ or ‘human’.

Fish: search items included: ‘fish’ or ‘seafood’ or ‘food’ and ‘diabetes’ or ‘diabetes mellitus’ or ‘T2DM’ and ‘population’ or ‘human’.

Egg: search items included: ‘egg’ or ‘food’ and ‘diabetes’ or ‘diabetes mellitus’ or ‘T2DM’ and ‘population’ or ‘human’.

Dairy: search items included: ‘dairy’ or ‘milk’ or ‘dairy product’ or ‘yogurt’ or ‘food’ and ‘diabetes’ or ‘diabetes mellitus’ or ‘T2DM’ and ‘population’ or ‘human’.

Soy: search items included: ‘soy’ or ‘legume’ or ‘soy product’ or ‘food’ and ‘diabetes’ or ‘diabetes mellitus’ or ‘T2DM’ and ‘population’ or ‘human’.

Eligible studies are selected by further manual scanning of all included studies and relevant reference lists.

### 2.2. Study Selection

All included studies should match the following criteria:(1) the study must have a cohort design; (2) the endpoint of the study was the incidence or mortality of the T2DM; (3) the study had to report risk ratios and the results had to be within a 95% CI in the paper (if this not clear, we requested this information from the authors); and (4) the study had to show the participants’ intake of dietary protein or other high-protein foods such as meat, fish, egg, dairy, soy.

### 2.3. Data Extraction

We extracted the following information from each publication we selected: first the author of the publication, publication year, the country where the study was conducted, the number of the samples and cases, T2DM diagnosis and criteria, the year the study began and finished, the years of follow-up, the methods of diet exposure assessment, the RRs and 95% CI, and the adjustment factors (Tables S1–S12).

### 2.4. Statistical Methods

We used effect models to calculate the summary RRs and 95% confidence to compare the highest dietary protein consumption with the lowest dietary protein consumption [19]. Two-sided *p* < 0.05 was considered statistically significant. The black square in the forest plots represents the weight contribution of every study. In order to evaluate the extent of variability, the *I*^2^-test statistic was adapted to estimate the heterogeneity [20].When *I*^2^ < 50% and *p* > 0.05, there was no heterogeneity; we used the fixed-effect model. The random-effect model was selected when *I*^2^ > 50% or *p* < 0.05. We used the Egger linear regression test and Begg rank correlation test to search for publication bias. When *p* > 0.05, there was no publication bias. Comprehensive Meta-Analysis V2 was employed for data analysis.

## 3. Results

### 3.1. Dietary Protein Intake and Risk of T2DM

**Figure 1:**
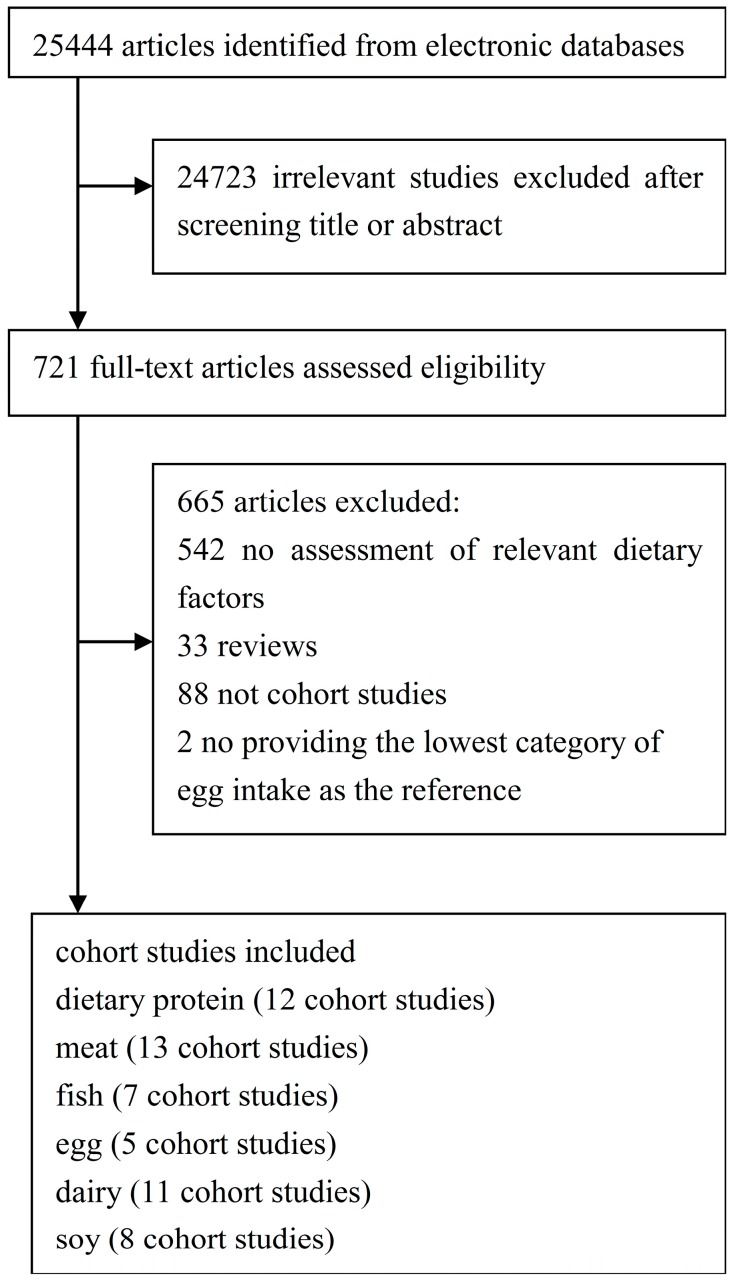
Flow chart for study selection.

We included 11 cohort studies in the analysis (Figure 1, Tables S1 and S2). Six of the studies were from the Unite States, three from Europe, one from Asia, one from Melboume, one from Finland.

**Figure 2:**
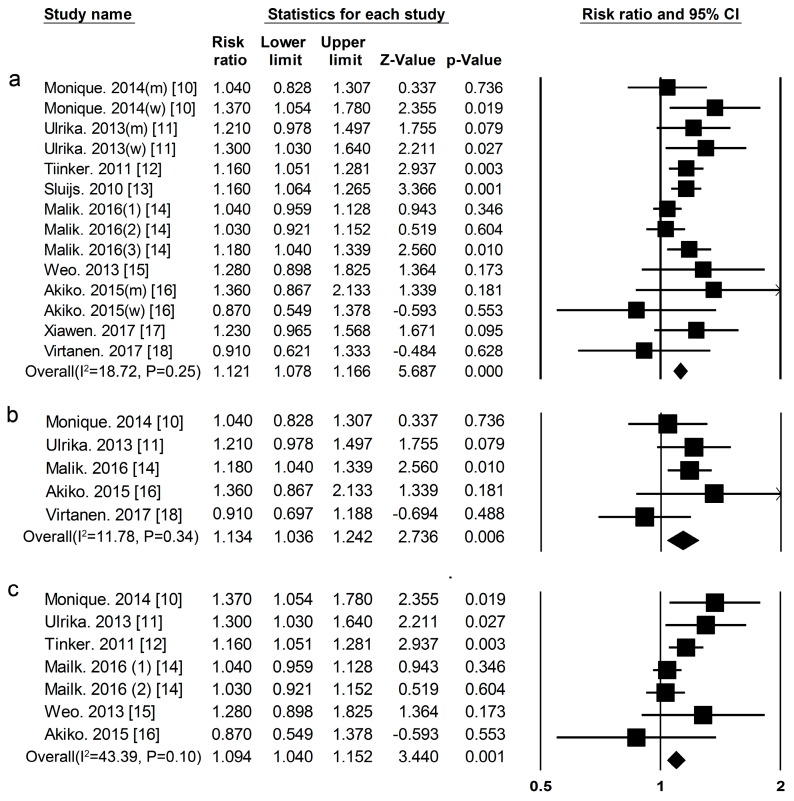
Total protein and type 2 diabetes relative risks(RRs) for (**a**) the highest vs. the lowest intake in all subjects and (**b**) the highest vs. the lowest intake in men (**c**) the highest vs. the lowest intake in women. The RR of each study is represented by a square, 95% CI are represented by the horizontal lines, and the diamond represents the estimate and its 95% CI.

***Total Protein*** Eleven cohort studies [10,11,12,13,14,15,16,17,18] researched the association between total protein intake and the risk of T2DM, included 52,637 cases among 483,174 participants. The summary RR and 95% CI for high vs. low values of all studies was 1.12 (1.08–1.17) (Figure 2a), (*I*^2^ = 18.72, *p* = 0.25) in all subjects, 1.13 (1.04–1.24) (Figure 2b) (*I*^2^ = 11.78, *p* = 0.34) in men, and 1.09(1.04–1.15) (Figure 2c) (*I*^2^ = 43.39, *p* = 0.10) in women.

**Figure 3:**
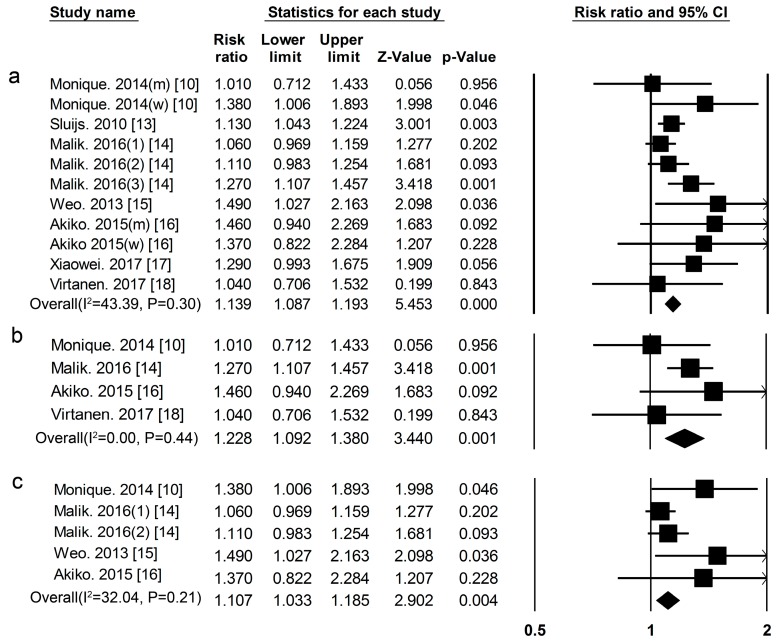
Animal protein and type 2 diabetes RRs for (**a**) the highest vs. the lowest intake in all subjects and (**b**) the highest vs. the lowest intake in men (**c**) the highest vs. the lowest intake in women. The RR of each study is represented by a square, 95% CI are represented by the horizontal lines, and the diamond represents the estimate and its 95% CI.

***Animal Protein*** Nine cohort studies [10,13,14,15,16,17,18] researched the association between animal protein intake and the risk of T2DM. This included 31,557 cases among 380,689 participants. The summary RR and 95% CI for high vs. low values of all studies was 1.14 (1.09–1.19) (Figure 3a), (*I*^2^ = 43.39, *p* = 0.30) in all subjects, 1.23 (1.09–1.38) (Figure 3b), (*I*^2^ = 0.00, *p* = 0.44) in men, and 1.11 (1.03–1.19) (Figure 3c), (*I*^2^ = 32.04, *p* = 0.21) in women.

**Figure 4:**
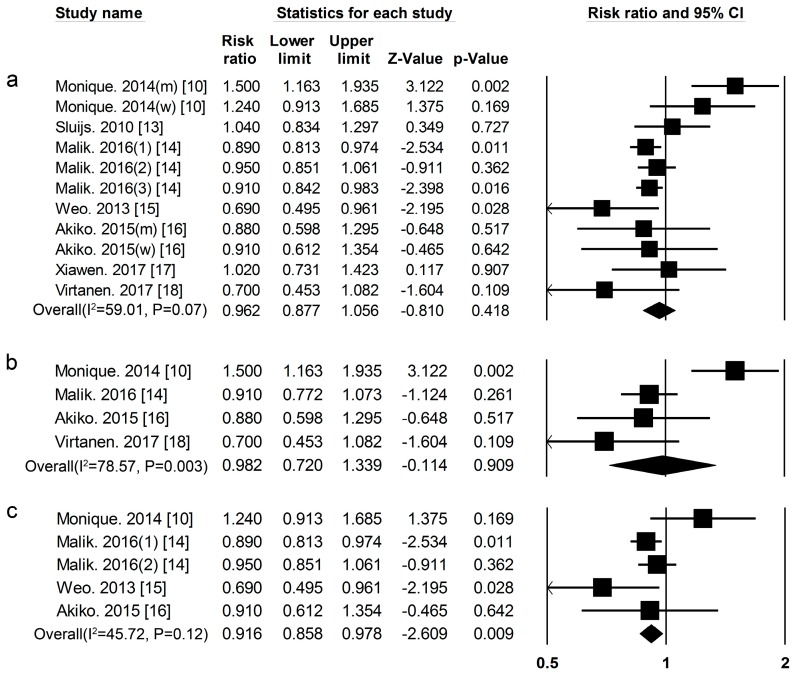
Plant protein and type 2 diabetes RRs for (**a**) the highest vs. the lowest intake in all subjects and (**b**) the highest vs. the lowest intake in men (**c**) the highest vs. the lowest intake in women. The RR of each study is represented by a square, 95% CI are represented by the horizontal lines, and the diamond represents the estimate and its 95% CI.

***Plant Protein*** Nine cohort studies [10,13,14,15,16,17,18] researched the association between plant protein and the risk of T2DM, included 31,817 cases among 381,879 participants. The summary RR and 95% CI for high vs. low values of all studies was 0.96 (0.88–1.06) (Figure 4a), (*I*^2^ = 59.01, *p* = 0.07) in all subjects. We used a sensitivity analysis to exclude the most influential studies: the summary RR and 95% CI ranged from 0.92 (0.87–0.98) when the European men’s study [10] was excluded to 0.98 (0.87–1.10) when the USA men’s study [14] was excluded. The heterogeneity was partly because of the European men’s study [10] and when we excluded this study, there was moderate heterogeneity (*I*^2^ = 14.85, *p* = 0.31). The summary RR and 95% CI was 0.98 (0.72–1.34) (Figure 4b) (*I*^2^ = 78.57, *p* = 0.003) in men. A sensitivity analysis was used to exclude the most influential studies: the summary RR and 95% CI ranged from 0.88 (0.76–1.02) when the European study [10] was excluded to 1.07 (0.75–1.52) when the Finnish men’s study [18] was excluded. The heterogeneity in men still existed after sensitivity analysis, partly because there were only four cohort studies about the association between plant protein intake and the risk of T2DM in men. The summary RR and 95% CI was 0.92 (0.86–0.97) (Figure 4c) (*I*^2^ = 45.72, *p* = 0.12) in women.

### 3.2. High-Protein Food and Risk of T2DM

We also conducted a systematic review and meta-analysis of cohort studies to clarify the association with high dietary protein food consumption and the risk of T2DM.

**Figure 5:**
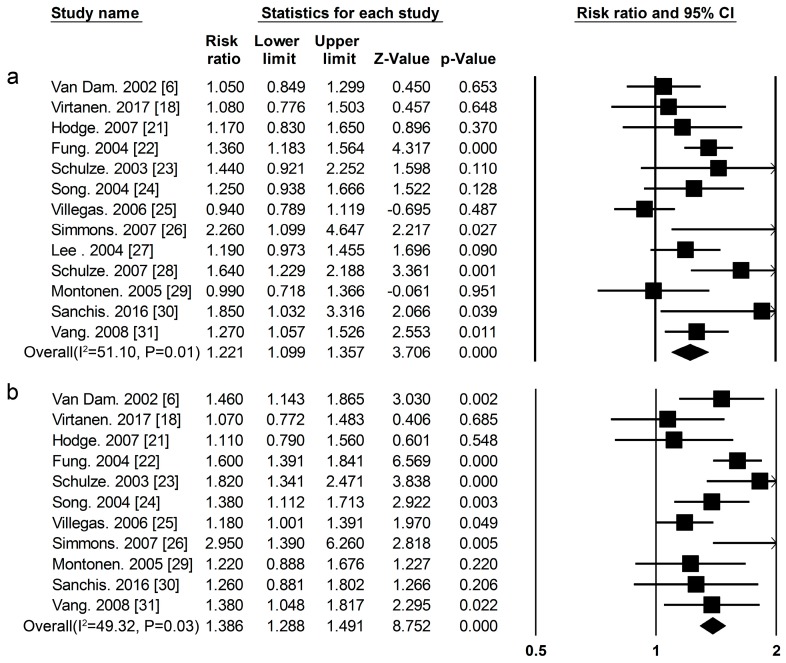
(**a**) Red meat (**b**) processed meat and type 2 diabetes RRs for the highest vs. the lowest intake in all subjects. The RR of each study is represented by a square, 95% CI are represented by the horizontal lines, and the diamond represents the estimate and its 95% CI.

***Red Meat*** Thirteen cohort studies [6,18,21,22,23,24,25,26,27,28,29,30,31] researched the association between red meat consumption and the risk of T2DM (Figure 1, Tables S3 and S4).The summary RR and 95% CI for high vs. low red meat consumption was 1.22 (1.09–1.36) (Figure 5a) (*I*^2^ = 51.11, *p* = 0.01). We used a sensitivity analysis to exclude the most influential studies: the summary RR and 95% CI ranged from1.20 (1.07–1.35) when the U.S. Study [24] was excluded to 1.26 (1.15–1.38) when the Chinese study [25] was excluded. The heterogeneity was partly because of the Chinese study [25], and when we excluded this study, there was moderate heterogeneity (*I*^2^ = 27.68, *p* = 0.17).

***Processed Meat*** Eleven cohort studies [6,18,21,22,23,24,25,26,29,30,31] researched the association between processed meat consumption and the risk of T2DM (Figure 1, Tables S3 and S4), the summary RR and 95% CI for high vs. low of processed meat consumption was 1.39 (1.29–1.49) (Figure 5b) (*I*^2^ = 49.32, *p* = 0.03).

**Figure 6:**
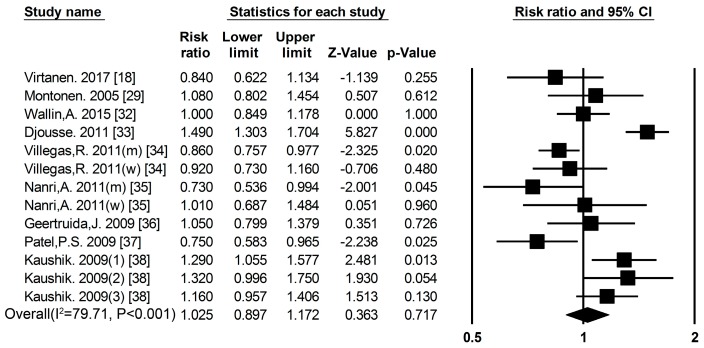
Fish and type 2 diabetes RRs for the highest vs. the lowest intake in all subjects. The RR of each study is represented by a square, 95% CI are represented by the horizontal lines, and the diamond represents the estimate and its 95% CI.

***Fish*** Nine cohort studies [18,29,32,33,34,35,36,37,38] researched the association between fish consumption and the risk of T2DM (Figure 1, Tables S5 and S6), the summary RR and 95% CI for high vs. low values of fish consumption was 1.03 (0.89–1.17) (Figure 6) (*I*^2^ = 79.71, *p* < 0.001). We used a sensitivity analysis to exclude the most influential studies: the summary RR ranged from0.99 (0.89–1.10) when the American study [33] was excluded to 1.50 (0.92–1.20) when the Japanese study [35] was excluded. The heterogeneity was partly because of the Japanese study [35], and when we excluded this study, there was moderate heterogeneity (*I*^2^ = 79.41, *p* *<* 0.001).

**Figure 7:**
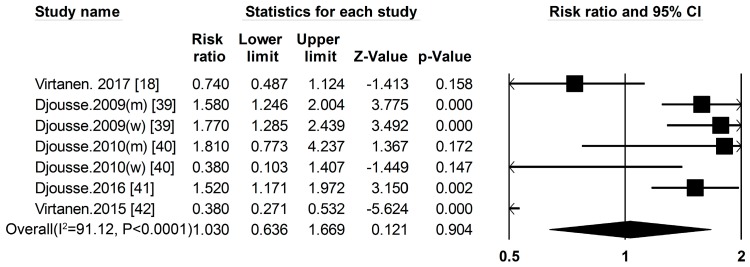
Egg and type 2 diabetes RRs for the highest vs. the lowest intake in all subjects. The RR of each study is represented by a square, 95% CI are represented by the horizontal lines, and the diamond represents the estimate and its 95% CI.

***Egg*** Five cohort studies [18,39,40,41,42] researched the association between egg consumption and the risk of T2DM (Figure 1, Tables S7 and S8), the summary RR and 95% CI for high vs. low of egg consumption was 1.03 (0.64–1.67) (Figure 7) (*I*^2^ = 91.12, *p* *<* 0.001). We used a sensitivity analysis to exclude the most influential studies: the summary RR ranged from 0.97 (0.49–1.88) when the Lithuanian study [39] was excluded to 1.57 (1.30–1.89) when the Finnish study [42] was excluded. The heterogeneity was partly because of the Finnish study [42], and when we excluded this study, there was moderate heterogeneity (*I*^2^ = 69.43, *p* = 0.06).

**Figure 8:**
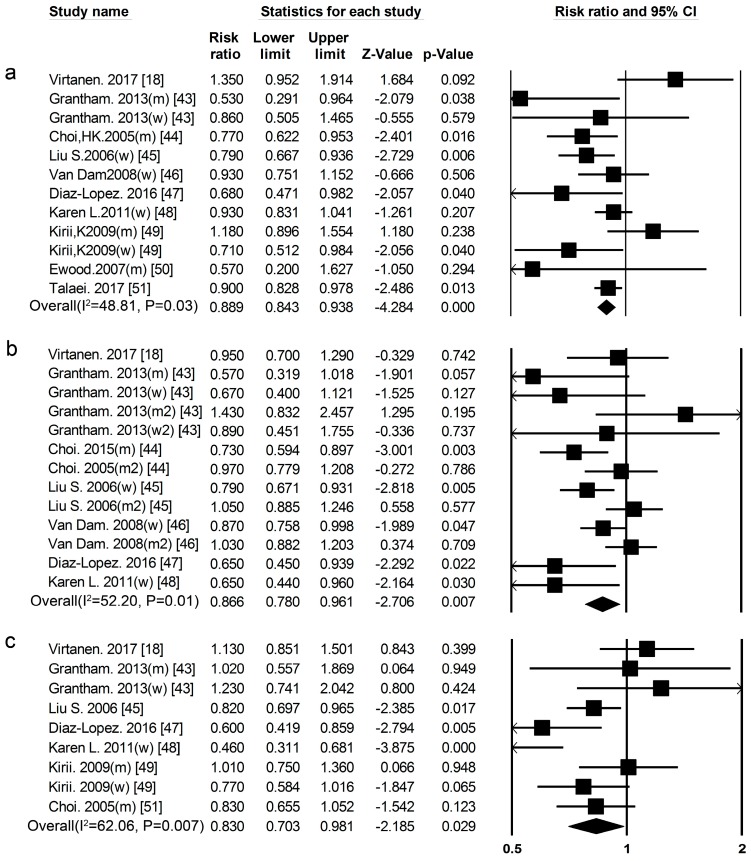
(**a**) Total dairy products (**b**) whole milk (**c**) yogurt and type 2 diabetes RRs for the highest vs. the lowest intake in all subjects. The RR of each study is represented by a square, 95% CI are represented by the horizontal lines, and the diamond represents the estimate and its 95% CI.

***TotalDairyProduct Consumption*** Eleven cohort studies [18,43,44,45,46,47,48,49,50,51,52] researched the association between total dairy product consumption and the risk of T2DM (Figure 1, Tables S9 and S10), the summary RR and 95% CI for high vs. low total dairy product consumption was 0.89 (0.84–0.94) (Figure 8a) (*I*^2^ = 48.81, *p* = 0.03). 

***Whole Milk*** Seven cohort studies [18,43,44,45,46,47,48] researched the association between whole milk consumption and risk of T2DM (Figure 1, Tables S9 and S10), the summary RR and 95% CI for high vs. low of fat dairy consumption was 0.87 (0.78–0.96) (Figure 8b) (*I*^2^ = 52.20, *p* = 0.01). We used a sensitivity analysis to exclude the most influential studies: the summary RR ranged from 0.85 (0.76–0.94) when the American study [46] was excluded to 0.89 (0.80–0.99) when the Japanese study [44] was excluded. The heterogeneity was partly because of the American study [46], and when we excluded this study, there was moderate heterogeneity (*I*^2^ = 46.87, *p* = 0.04).

***Yogurt*** Seven cohort studies [18,43,44,47,48,49] researched the association between yogurt consumption and the risk of T2DM (Figure 1, Tables S9 and S10).The summary RR for high vs. low yogurt consumption was 0.83 (0.70–0.98) (Figure 8c) (*I*^2^ = 62.06, *p* = 0.01). We used a sensitivity analysis to exclude the most influential studies: the summary RR ranged from 0.81 (0.67–0.97) when the Japanese study [49] was excluded to 0.88 (0.76–1.00) when USA study [48] was excluded. The heterogeneity was partly because of the Japanese study [49], and when we excluded this study there was moderate heterogeneity (*I*^2^ = 40.61, *p* = 0.11).

**Figure 9:**
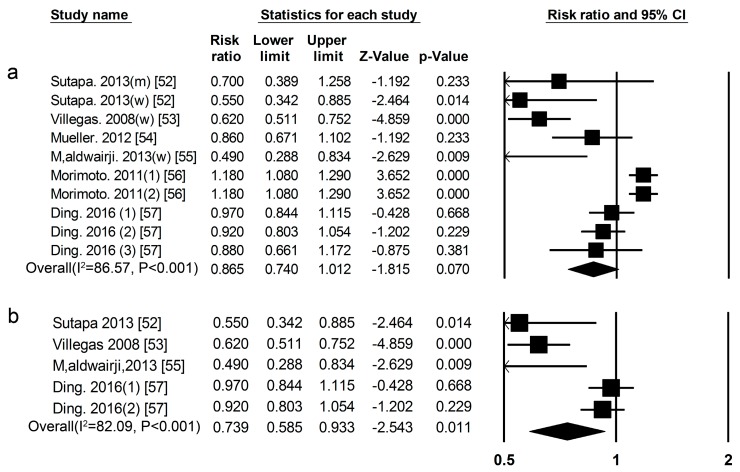
Soy and type 2 diabetes RRs for (**a**) the highest vs. the lowest intake in all subjects and (**b**) the highest vs. the lowest intake in women. The RR of each study is represented by a square, 95% CI are represented by the horizontal lines, and the diamond represents the estimate and its 95% CI.

***Soy*** Eight cohort studies [52,53,54,55,56,57] researched the association between legume consumption and the risk of T2DM (Figure 1, Tables S11 and S12), the summary RR for high vs. low soy consumption was 0.87 (0.74–1.01) (Figure 9a) (*I*^2^ = 86.57, *p* < 0.01). We used a sensitivity analysis to exclude the most influential studies: the summary RR ranged from0.82 (0.68–0.98) when the American study [56] was excluded to 0.93 (0.81–1.06) when the Chinese study [53] was excluded. The heterogeneity was partly because of the American study [56], and when we excluded this study, there was moderate heterogeneity (*I*^2^ = 69.34, *p* < 0.01). However, the summary RR for high vs. low soy was 0.74 (0.59–0.93) (Figure 9b), (*I*^2^ = 82.09, *p* < 0.001) in women. We used a sensitivity analysis to exclude the most influential studies: the summary RR ranged from0.66 (0.49–0.90) when the American study [57] was excluded to 0.81 (0.65–1.00) when the Chinese study [54] was excluded. The heterogeneity was partly because of the American study [57], and when we excluded this study, there was moderate heterogeneity (*I*^2^ = 0.00, *p* = 0.67).

### 3.3. Publication Bias

No significant publication bias was detected in the Begg-Mazumdar’s test and Egger’s test of our meta-analyses, provided in Table 1.

## 4. Discussion

According to the results of this meta-analysis, the intake of total protein and animal protein was associated with a high risk of T2DM both in males and females. The intake of plant protein was associated with low risk of T2DM in females, but not in males. In high animal protein food, red meat, and processed meat were associated with a high risk of T2DM in all subjects, while total dairy products, low-fat dairy, and yogurt were associated with a low risk of T2DM in all subjects, and egg and fish were not associated with a decreased risk of T2DM. In high plant protein food, soy was associated with a low risk of T2DM in females.

Higher intake of dietary protein is often associated with lifestyles, including physical activity, body weight, smoking, drinking. For example, we already know that overweight and obesity are risk factors for T2DM, and a meta-analysis showed that each unit increase of BMI would increase the risk for T2DM by approximately 20% [58]. In our meta-analysis, most studies were adjusted for known influencing factors, including age, BMI (Body Mass Index), smoking, physical activity, alcohol consumption, energy intake, family history of T2DM and menopausal status (among women).

The statistical power of the results could be significantly increased as the number of studies and the sample size increase, but it could also lead to heterogeneity. Some heterogeneity was due to different participants’ characteristics and regions, and different dietary assessment methods. Thus, heterogeneity is usually used to explain the study characteristics and is difficult to interpret. In our study, the heterogeneities of total protein and animal protein were in the acceptable range, but the heterogeneity of plant protein was outside of the range. We found that the European study [10] contributed to the heterogeneity. When this study was excluded, the heterogeneities of both the overall and subgroup analyses were much lower. The reason that the European study [10] had inconsistent results compared with other studies was not clear, but it could partly be due to the participants in this study having a lower plant protein intake than other studies [13,14,15,16,17,18]. For red meat and processed meat, we found that the Chinese study [25] contributed to the heterogeneity. When this study is excluded, the heterogeneities were much lower. The reason that the Chinese study [25] had inconsistent results may be due to the participants in this study having a lower meat intake than other participants [6,18,21,22,23,24,26,27,28,29,30,31]. For fish, we found that the Japanese study [35] contributed to the heterogeneity, but the heterogeneity was not moderate when it was excluded, which means the association between fish and T2DM needs further refinement. For egg, we found that the Finnish study [42] contributed to the heterogeneity. When this study is excluded, the heterogeneity was much lower. The reason why the Finnish study [42] had inconsistent results compared with other studies was not clear, but it could partly be due to the participants in this study being older than the other studies [18,39,40,41]. For whole milk, we found that the American study [46] contributed to the heterogeneity, and the heterogeneity was moderate when it was excluded. The reason why the American study [46] had heterogeneity may be due to the milk intake of black women in America being different from the participants of other studies’ [18,43,44,45,47,48]. For yogurt, the Japanese study [49] contributed to the heterogeneity. The reason why the Japanese study [49] had inconsistent results was not clear, but it could partly be due to the female participants in this study being older than other studies [18,43,44,47,48].

As meta-analysis is based on published studies, the publication bias affect is inevitable. It is particularly important to evaluate publication bias. In our meta-analysis, we used the Egger linear regression test and Begg rank correlation test to determine publication bias, and we found that there was no publication bias in our study.

Dietary protein and amino acids are involved in the modulations of insulin sensitivity and glucose metabolism. However, the results from human studies were still inconsistent. Some studies showed that high intake of dietary protein had negative effects on glucose homeostasis by facilitating insulin resistance and increasing gluconeogenesis [7,59,60]. Amino acid signaling may facilitate insulin resistance, by activation of the mammalian target of rapamycin (mTOR), a nutrient sensor that operates a detrimental feedback loop toward insulin receptor substrate 1 signaling [61,62,63,64]. Moreover, amino acids may also inhibit glucose uptake through phosphorylation of downstream factors of the insulin signaling cascade by the translation initiation factor serine-kinase-6-1 [63,65]. On the other hand, in vivo and in vitro studies also demonstrated that amino acids play a beneficial role in glucose homeostasis by modulating insulin action on hepatic glucose production and muscle glucose transport, secretion of glucagon and insulin, as well as various tissues gene and protein expression [64,66]. One of the possible mechanisms that might explain this was that higher protein reduced the amount of carbohydrate intake under isoenergy conditions and thus a smaller amount of glucose was absorbed after ingestion of the meals, with the consequence of a reduced store of glycogen and, thus, a decrease in glycogenolysis rate [67,68]. The other possible causal mechanism was amino acids stimulating the insulin secretion to intervene in the glucose metabolism and serve as substrates for gluconeogenesis; thus, increased gluconeogenesis could stimulate insulin secretion, which might prevent hyperglycemia [69].Additionally, some scientists think that different qualities rather than quantities of proteins play a more important role in insulin resistance [69]. Our study supported the hypothesis that animal proteins caused a high risk of T2DM in males and females, and plant proteins were protection factors of T2DM in females. We can find some support for this from the literature. The abundance in certain amino acids are different between animal proteins and plant proteins. This may contribute to the different effects between them on the risk of T2DM. Typically, plant protein contains lower levels of the branched chain amino acids leucine, isoleucine and valine and of the sulfur amino acid methionine as compared with animal proteins [70]. Branched chain amino acids and higher methionine intake have been associated with insulin resistance and type 2 diabetes [71,72]. In addition, dietary glycine is also mainly consumed from animal-based foods and some cohort studies have shown that glycine was positively associated with T2DM, and hypertension [72,73]. On the other hand, dietary glutamic acid, an amino acid that is mainly consumed from plant protein was found to be inversely associated with risks of hypertension and arterial stiffness [73,74]. So far, three intervention studies have compared the effects of animal protein with plant protein meals on glycaemic variables in people with T2DM, but they were obtained from three different results [75,76,77]. This should be further investigated in future studies.

From the current literature available, the inconsistency in association between plant protein and T2DM was probably due to gender difference. The negative association between plant protein or soy and T2DM was observed mainly in women, while most of the studies in men found null results. Therefore, results from all subjects without considering gender difference were different and the proportion of women in the study may influence the results. However, the exact mechanism is unclear.

In order to further refine and compare the association between different food sources of protein and T2DM, and facilitate dietary guidance, we have analyzed the relationship between different high-protein food and the risk of type 2 diabetes. We found that different high-protein foods play different roles in T2DM, even if they are all animal-based foods. Our results indicated that the intake of red meat and processed meat are risk factors for T2DM. They are also positively associated with weight gain [78], stroke [79], coronary heart disease [80] and mortality [81].First, the increased meat protein may increase iron load, which was associated with the increased risk of T2DM [82]. Moreover, the other nutrients in red and processed meat, including nitrites and advanced glycation end products, were also thought to mediate the association between meat intake and the risk of T2DM [83].The relationship between egg consumption and T2DM was not clear. Some studies have shown that egg intake was associated with a lower risk of T2DM [42].Some research showed that egg consumption was positively associated with the risk of T2DM in our study [39,84,85], and this result was supported by the AHA dietary guidelines which advise restricted egg consumption in adults for preventing cardiometabolic diseases [86,87,88].We found that total dairy products, whole milk, and yogurt intake were protective factors for T2DM. Some studies showed that milk proteins, like whey protein, may enhance satiety and reduce risk factors for T2DM [89]. The calcium and vitamin D in milk and its products may also contribute to its beneficial effects on T2DM [90].In our study, fish consumption was not associated with decreased risk of T2DM. This result may partly relate on the increase in plasma selenium level with the increment of fish intake, which may increase the risk of diabetes [91,92].The relationship between fish intake and the risk of T2DM needs further refinement. For high plant protein-based foods, there was a negative association between soy consumption and the risk of T2DM in this study. Soy protein may inhibit insulin secretion from pancreatic β cells or inhibit lipogenesis and enhance lipolysis in the adipose and liver to reduce adiposity [93].This protective effect may also be associated with biologically active ingredients such as phytoestrogen in soybeans [94].

Our meta-analysis also had limitations. First, publications into our research were adjusted for BMI, but some studies had measurement errors because of self-reporting of height and weight, resulting in the BMI relying on self-reporting, which could lead to confounding results. Second, some important factors that influence T2DM such as fiber, lipids, and carbohydrates, were only adjusted in some of these studies, which may also lead to confounding results. Additionally, limitations might be due to temporal bias. Studies with longer follow-up might beless influenced by temporal bias. In our studies, the follow-up period of each research study was different, so the temporal bias might impact the association between dietary protein intake and the risk of T2DM.

## 5. Conclusions

In summary, we found that total protein and animal protein consumption were the risk factors for T2DM, and plant protein was the protective factor for T2DM in women, but not in men. We also found that different high-protein foods have a different effect on T2DM risk, even if they all belong to animal proteins. These results underline the significance of taking into account what kind of dietary protein and food sources of protein are recommended for the prevention of diabetes.

## Figures and Tables

**Table 1:** Public bias and meta-analysis.

Proteins and Foods Sources	Begg-Mazumdar’s Test	Egger’s Test
Total protein (all)	0.74	0.38
Total protein (men)	0.46	0.74
Total protein (women)	0.55	0.33
Animal protein (all)	0.64	0.07
Animal protein (men)	0.73	0.57
Animal protein (women)	0.22	0.10
Plant protein (all)	0.76	0.50
Plant protein (men)	0.73	0.90
Plant protein (women)	0.81	0.91
Red meat	0.30	0.33
Processed meat	0.88	0.99
Fish	0.58	0.43
Total dairy product	0.45	0.43
Whole milk	0.67	0.35
Yogurt	0.92	0.99
Egg	0.23	0.50
Soy	0.46	0.13

## Supplementary Materials

The following are available online at www.mdpi.com/2072-6643/9/9/982/s1, Table S1: Characteristics of participants and follow-up in included studies of protein consumption in relation to the risk of T2DM, Table S2: Prospective cohort studies of protein consumption and type 2 diabetes, Table S3: Characteristics of participants and follow-up in included studies of meat consumption in relation to the risk of T2DM, Table S4: Prospective cohort studies of meat consumption and type 2 diabetes, Table S5: Characteristics of participants and follow-up in included studies of fish consumption in relation to the risk of T2DM, Table S6: Prospective cohort studies of fish consumption and type 2 diabetes, Table S7: Characteristics of participants and follow-up in included studies of egg consumption in relation to risk of T2DM, Table S8:Prospective cohort studies of egg consumption and type 2 diabetes, Table S9: Characteristics of participants and follow-up in included studies of dairy consumption in relation to the risk of T2DM, Table S10: Prospective cohort studies of dairy consumption and type 2 diabetes, Table S11: Characteristics of participants and follow-up in included studies of soy consumption in relation to the risk of T2DM, Table S12: Prospective cohort studies of soy consumption and type 2 diabetes.

## References

[1] Guariguata L., Whiting D.R., Hambleton I., Beagley J., Linnenkamp U., Shaw J.E. (2014). Globalestimates of diabetes prevalence for 2013 and projections for 2035. Diabetes Res. Clin. Pract..

[2] American Diabetes Association (2006). Standards of medical care in diabetes—2006. Diabetes Care.

[3] Nichols G.A., Glauber H.S., Brown J.B. (2002). Type 2 diabetes: Incremental medical care costs during the 8 years preceding diagnosis. Diabetes Care.

[4] Tuomilehto J., Lindström J., Eriksson J.G., Valle T.T., Hämäläinen H., Ilanne-Parikka P., Keinänen-Kiukaanniemi S., Laakso M., Louheranta A., Rastas M. (2011). Prevention of: Type 2 diabetes mellitus by changes in lifestyle among subjects with impaired glucose tolerance. N. Engl. J. Med..

[5] Schulze M.B., Schulz M., Heidemann C., Schienkiewitz A., Hoffmann K., Boeing H. (2008). Carbohydrate intake and incidence of type 2 diabetes in the European Prospective Investigation into Cancer and Nutrition (EPIC)-Potsdam Study. Br. J. Nutr..

[6] Van Dam R.M., Willett W.C., Rimm E.B., Stampfer M.J., Hu F.B. (2002). Dietary fat and meat intake in relation to risk of type 2 diabetes in men. Diabetes Care.

[7] Weickert M.O., Roden M., Isken F., Hoffmann D., Nowotny P., Osterhoff M., Blaut M., Alpert C., Gögebakan Ö., Bumke-Vogt C. (2011). Effects of supplemented isoenergetic diets differing in cereal fiber and protein content on insulin sensitivity in overweight humans. Am. J. Nutr..

[8] Nuttal F.Q., Schweim K., Hoover H., Gannon M.C. (2008). Effect of the LoBAG30 diet on blood glucose control in people with type 2 diabetes. Br. J. Nutr..

[9] Pounis G.D., Tyrovolas S., Antonopoulou M., Zeimbekis A., Anastasiou F., Bountztiouka V., Metallinos G., Gotsis E., Lioliou E., Polychronopoulos E. (2010). Long-term animal-protein consumption is associated with an increased prevalence of diabetes among the elderly: The Mediterranean islands (MEDIS) study. Diabetes Metab..

[10] Van Nielen M., Feskens E.J., Mensink M., Sluijs I., Molina E., Amiano P., Ardanaz E., Balkau B., Beulens J.W., Boeing H. (2014). Dietary protein intake and incidence of type 2 diabetes in Europe: The EPIC-InterAct Case-Cohort Study. Diabetes Care.

[11] Ericson U., Sonestedt E., Gullberg B., Hellstrand S., Hindy G., Wirfalt E., Orho M. (2013). High intakes of protein and processed meat associate with increased incidence of type 2 diabetes. Br. J. Nutr..

[12] Tinker L.F., Sarto G.E., Howard B.V., Huang Y., Neuhouser M.L., Mossavar-Rahmani Y., Beasley J.M., Margolis K.L., Eaton C.B., Phillips L.S. (2011). Biomarker-calibrated dietary energy and protein intake associations with diabetes risk among postmenopausal women from the Women’s Health Initiative. Am. J. Clin. Nutr..

[13] Sluijs I., Beulens J.W., Spijkerman A.M., Grobbee D.E., van der Schouw Y.T. (2010). Dietary intake of total, animal, and vegetable protein and risk of type 2 diabetes in the European Prospective Investigation into Cancer and Nutrition(EPIC)-NL study. Diabetes Care.

[14] Malik V., Li Y., Tobias D., Pan A., Hu F. (2016). Dietary protein intake and risk of type 2 diabetes in U.S. men and women. Am. J. Epidemiol..

[15] Bao W., Bowers K., Tobias D., Hu F., Zhang C. (2013). Prepregnancy dietary protein intake, major dietary protein sources, and the risk of gestational diabetes mellitus: A Prospective cohort study. Diabetes Care.

[16] Nanri A., Mizoue T., Kurotani K., Goto A., Oba S., Noda M., Sawada N., Tsugane S. (2015). Low-carbohydrate diet and type 2 diabetes risk in Japanese men and women: The Japan Public Health Center-Based Prospective Study. PLoS ONE.

[17] Shang X., Scott D., Hodge A.M., English D.R., Giles G.G., Ebeling P.R., Sanders K.M. (2016). Dietary protein intake and risk of type 2 diabetes: Results from Melboune Collaborative Cohort study and a meta-analysis of prospective studies. Am. J. Clin. Nutr..

[18] Virtanen H.E., Koskinen T.T., Voutilainen S., Mursu J., Tuomainen T.P., Kokko P., Virtanen J.K. (2017). Intake of different dietary proteins and risk of type 2 diabetes in men: The Kuopio Ischaemic Heart Disease Risk Factor Study. Br. J. Nutr..

[19] Der Simonian R., Laird N. (1986). Meta-analysis in clinical trials. Control Clin. Trials.

[20] Higgins J.P., Thompson S.G. (2002). Quantifying heterogeneity in a meta-analysis. Stat. Med..

[21] Hodge A.M., English D.R., O’Dea K., Giles G.G. (2007). Dietary patterns and diabetes incidence in the Melbourne Collaborative Cohort Study. Am. J. Epidemiol..

[22] Fung T.T., Schulze M., Manson J.E., Willett W.C., Hu F.B. (2004). Dietary patterns, meat intake, and the risk of type 2 diabetes in women. Arch. Intern. Med..

[23] Schulze M.B., Manson J.E., Willett W.C., Hu F.B. (2003). Processed meat intake and incidence of type 2 diabetes in younger and middle-aged women. Diabetologia.

[24] Song Y., Manson J.E., Buring J.E., Liu S. (2004). A prospective study of red meat consumption and type 2 diabetes in middle-aged and elderly women: The women’s health study. Diabetes Care.

[25] Villegas R., Shu X.O., Gao Y.T., Yang G., Cai H., Li H., Zheng W. (2006). The association of meat intake and the risk of type 2 diabetes may be modified by body weight. Int. J. Med. Sci..

[26] Simmons R.K., Harding A.H., Wareham N.J., Griffin S.J. (2007). Do simple questions about diet and physical activity help to identify those at risk of type 2 diabetes?. Diabet. Med..

[27] Lee D.H., Folsom A.R., Jacobs D.R. (2004). Dietary iron intake and type 2 diabetes incidence in postmenopausal women: The Iowa Women’s Health Study. Diabetologia.

[28] Schulze M.B., Hoffmann K., Boeing H., Linseisen J., Rohrmann S., Möhlig M., Pfeiffer A.F., Spranger J., Thamer C., Häring H.U. (2007). An accurate risk score based on anthropometric, dietary, and lifestyle factors to predict the development of type 2 diabetes. Diabetes Care.

[29] Montonen J., Jarvinen R., Heliovaara M., Reunanen A., Aromaa A., Knekt P. (2005). Food consumption and the incidence of type II diabetes mellitus. Eur. J. Clin. Nutr..

[30] Mari-Sanchis A., Gea A., Basterra-Gortari F.J., Martinez-Gonzalez M.A., Beunza J.J., Bes-Rastrollo M. (2016). Meat consumption and risk of developing type 2 diabetes in the SUN Project: A highly educated middle-class population. PLoS ONE.

[31] Vang A., Singh P.N., Lee J.W., Haddad E.H., Brinegar C.H. (2008). Meats, processed meats, obesity, weight gain and occurrence of diabetes among adults: Findings from adventist health studies. Ann. Nutr. Metab..

[32] Wallin A., Di Giuseppe D., Orsini N., Åkesson A., Forouhi N.G., Wolk A. (2017). Fish consumption and frying of fish in relation to type 2 diabetes incidence: A prospective cohort study of Swedish men. Eur. J. Nutr..

[33] Djoussé L., Gaziano J.M., Buring J.E., Lee I.M. (2011). Dietary omega-3 fatty acids and fish consumption and risk of type 2 diabetes. Am. J. Clin. Nutr..

[34] Villegas R., Xiang Y., Elasy T., Li H., Yang G., Cai H., Ye F., Gao Y., Shyr Y., Zheng W. (2011). Fish, shellfish, and long-chain *n*-3 fatty acid consumption and risk of incident type 2 diabetes in middle-aged Chinese men and women. Am. J. Clin. Nutr..

[35] Nanri A., Mizoue T., Noda M., Takahashi Y., Poudel T., Kato M., Oba S., Inoue M., Tsugane S. (2011). Fish intake and type 2 diabetes in Japanese men and women: The Japan Public Health Center-based Prospective Study. Am. J. Clin. Nutr..

[36] Geertruida J., Ballegooijen A., Kuijsten A., Sijbrands E., Rooij F., Geleijnse J., Hofman A., Witteman J., Fesken E. (2009). Eating fish and risk of type 2 diabetes: A population-based, prospective follow-up study. Diabetes Care.

[37] Patel P., Sharp S., Luben R., Khaw K., Bingham S., Wareham N., Forouhi N. (2009). Association between type of dietary fish and seafood intake and the risk of incident type 2 diabetes: The European prospective investigation of cancer (EPIC)-Norfolk cohort study. Diabetes Care.

[38] Kaushik M., Mozaffarian D., Spiegelman D., Manson J.E., Willett W.C., Hu F.B. (2009). Long chain omega-3 fatty acids, fish intake, and the risk of type 2 diabetes mellitus. Am. J. Clin. Nutr..

[39] Djousse L., Gaziano J.M., Buring J.E., Lee I.M. (2009). Egg consumption and risk of type 2 diabetes in men and women. Diabetes Care.

[40] Djoussé L., Kamineni A., Nelson T.L., Carnethon M., Mozaffarian D., Siscovick D., Mukamal K.J. (2010). Egg consumption and risk of type 2 diabetes in older adults. Am. J. Clin. Nutr..

[41] Djousse L., Petrone A., Hickson D., Talegawkar S., Dubbert P., Taylor H., Tucker K. (2016). Egg consumption and risk of type 2 diabetes among African Americans: The Jackson Heart Study. Clin. Nutr..

[42] Virtanen J., Mursu J., Tuomainen T., Virtanen H., Voutilainen S. (2015). Egg consumption and risk of incident type 2 diabetes in men: The Kuopio Ischaemic Heart Disease Risk Factor Study. Am. J. Clin. Nutr..

[43] Grantham N., Magliano D., Hodge A., Jowett J., Meikle P., shaw J. (2013). The association between dairy food intake and the incidence of diabetes in Australia: The Australian Diabetes Obesity and Lifestyle Study (AusDiab). Public Health Nutr..

[44] Choi H.K., Willett W.C., Stampfer M.J., Rimm E., Hu F.B. (2005). Dairy consumption and risk of type 2 diabetes mellitus in men: A prospective study. Arch. Intern. Med..

[45] Liu S., Choi H.K., Ford E., Song Y., Klevak A., Buring J.E., Manson J.E. (2006). A prospective study of dairy intake and the risk of type 2 diabetes in women. Diabetes Care.

[46] Van Dam R.M., Hu F.B., Rosenberg L., Krishnan S., Palmer J.R. (2006). Dietary calcium and magnesium, major food sources, and risk of type 2 diabetes in U.S. black women. Diabetes Care.

[47] Díaz-López A., Bulló M., Martínez-González M.A., Corella D., Estruch R., Fitó M., Gómez-Gracia E., Fiol M., de la Corte F.J.G., Ros E. (2016). Dairy product consumption and risk of type 2 diabetes in an elderly Spanish Mediterranean population at high cardiovascular risk. Eur. J. Nutr..

[48] Margolis K.L., Wei F., de Boer I.H., Howard B.V., Liu S., Manson J.E., Mossavar-Rahmani Y., Phillips L.S., Shikany J.M., Tinker L.F. (2011). A diet high in low-fat dairy products lowers diabetes risk in postmenopausal women. J. Nutr..

[49] Kirii K., Mizoue T., Iso H., Takahashi Y., Kato M., Inoue M., Noda M., Tsugane S., Japan Public Health Center-based Prospective Study Group (2009). Calcium, vitamin D and dairy intake in relation to type 2 diabetes risk in a Japanese cohort. Diabetologia.

[50] Elwood P.C., Pickering J.E., Fehily A.M. (2007). Milk and dairy consumption, diabetes and the metabolic syndrome: The Caerphilly prospective study. J. Epidemiol. Community Health.

[51] Talaei M., Pan A., Yuan J.M., Koh W.P. (2017). Dairy intake and risk of type 2 diabetes. Clin. Nutr..

[52] Sutapa A., Shah E. (2013). Association between legume intake and self-reported diabetes among adult men and women in India. BMC Public Health.

[53] Villegas R., Gao Y., Yang G., Li H., Elasy T., Zheng W., Shu X. (2008). Legume and soy food intake and the incidence of type 2 diabetes in the Shanghai Women’s Health Study. Am. J. Clin. Nutr..

[54] Mueller N., Odegaard A., Gross M., Koh W., Yu M., Yuan J., Pereira M. (2012). Soy intake and risk of type 2 diabetes in Chinese Singaporeans. Eur. J. Nutr..

[55] Aldwairji M., Orfila C., Burley V.J. (2013). Legume intake and risk of type 2 diabetes in British women. Proc. Nutr. Soc..

[56] Morimoto Y., Steinbrecher A., Kolonel L., Maskarinec G. (2011). Soy consumption is not protective against diabetes in Hawaii: The Multiethnic Cohort. Eur. J. Clin. Nutr..

[57] Ding M., Pan A., Manson J.E., Willett W.C., Malik V., Rosner B., Giovannucci E., Hu F.B., Sun Q. (2016). Consumption of soy foods and isoflavones and risk of type 2diabetes: A pooled analysis of three U.S. cohorts. Eur. J. Clin. Nutr..

[58] Hartemink N., Boshuizen H.C., Nagelkerke N.J., Jacobs M.A., van Houwelingen H.C. (2006). Combining risk estimates from observational studies with different exposure cutpoints: A meta-analysis on body mass index and diabetes type 2. Am. J. Epidemiol..

[59] Linn T., Geyer R., Prassek S., Laube H. (1996). Effect of dietary protein intake on insulin secretion and glucose metabolism in insulin-dependent diabetes mellitus. J Clin. Endocrinol. Metab..

[60] Linn T., Santosa B., Grönemeyer D., Aygen S., Scholz N., Busch M., Bretzel R.G. (2000). Effect of long-term dietary protein intake on glucose metabolism in humans. Diabetologia.

[61] Patti M.E., Brambilla E., Luzi L., Landaker E.J., Kahn C.R. (1998). Bidirectional modulation of insulin action by amino acids. J. Clin. Investig..

[62] Takano A., Usui I., Haruta T., Kawahara J., Uno T., Iwata M., Kobayashi M. (2001). Mammalian target of rapamycin pathway regulates insulin signaling via subcellular redistribution of insulin receptor substrate 1 and integrates nutritional signals and metabolic signals of insulin. Mol. Cell. Biol..

[63] Tremblay F., Krebs M., Dombrowski L., Brehm A., Bernroider E., Roth E., Nowotny P., Waldhäusl W., Marette A., Roden M. (2005). Overactivation of S6 kinase 1 as a cause of human insulin resistance during increased amino acid availability. Diabetes.

[64] Tremblay F., Marette A. (2001). Amino acid and insulin signaling via the mTOR/p70 S6 kinase pathway. A negative feedback mechanism leading to insulin resistance in skeletal muscle cells. J. Biol. Chem..

[65] Um S.H., D’Alessio D., Thomas G. (2006). Nutrient overload, insulin resistance, and ribosomal protein S6 kinase 1, S6K1. Cell Metab..

[66] Gulve E.A., Cartee G.D., Holloszy J.O. (1991). Prolonged incubation of skeletal muscle in vitro: Prevention of increases in glucose transport. Am. J. Physiol..

[67] Gannon M.C., Nuttall F.Q. (2004). Effect of a high-protein, low-carbohydrate diet on blood glucose control in people with type 2 diabetes. Diabetes.

[68] Nuttall F.Q., Gannon M.C. (2004). Metabolic response of people with type 2 diabetes to a high-protein diet. Nutr. Metab..

[69] Tremblay F., Lavigne C., Jacques H., Marette A. (2007). Role of dietary proteins and amino acids in the pathogenesis of insulin resistance. Annu. Rev. Nutr..

[70] Richter C.K., Skulas-Ray A.C., Champagne C.M., Kris-Etherton P.M. (2015). Plantprotein and animal proteins: Do they differentially affect cardiovascular disease risk?. Adv. Nutr..

[71] Newgard C.B. (2012). Interplay between lipids and branched-chain amino acids in development of insulin resistance. Cell Metab..

[72] Wittenbecher C., Muhlenbruch K., Kroger J., Jacobs S., Kuxhaus O., Floegel A., Fritsche A., Pischon T., Prehn C., Adamski J. (2015). Amino acids, lipid metabolites, and ferritin as potential mediators linking red meat consumption to type 2 diabetes. Am. J. Clin. Nutr..

[73] Stamler J., Brown I.J., Daviglus M.L., Chan Q., Miura K., Okuda N., Ueshima H., Zhao L., Elliott P. (2013). Dietary glycine and blood pressure: The international study on macro/micronutrients and blood pressure. Am. J. Clin. Nutr..

[74] Jennings A., MacGregor A., Welch A., Chowienczyk P., Spector T., Cassidy A. (2015). Amino acid intakes are inversely associated with arterial stiffness and central blood pressure in women. J. Nutr..

[75] Azadbakht L., Atabak S., Esmaillzadeh A. (2008). Soy protein intake, cardiorenal indices, and C-reactive protein in type 2 diabetes with nephropathy: A longitudinal randomized clinical trial. Diabetes Care.

[76] Wheeler M.L., Fineberg S.E., Fineberg N.S., Gibson R.G., Hackward L.L. (2002). Animal versus plant protein meals in individuals with type 2 diabetesand microalbuminuria: Effects on renal, glycemic, and lipid parameters. Diabetes Care.

[77] Sucher S., Markova M., Hornemann S., Pivovarova O., Rudovich N., Thomann R., Schneeweiss R., Rohn S., Pfeiffer A.F. (2017). Compar ison of the effects of diets high in animal or plantprotein on metaboli c and cardiov ascular markers in type 2diabetes: A randomize clinical trial. Diabetes Obes. Metab..

[78] Mozaffarian D., Hao T., Rimm E.B., Willett W.C., Hu F.B. (2011). Changes in diet and lifestyle and long-term weight gain in women and men. N. Engl. J. Med..

[79] Bernstein A.M., Pan A., Rexrode K.M., Stampfer M., Hu F.B., Mozaffarian D., Willett W.C. (2012). Dietary protein sources and the risk of stroke in men and women. Stroke.

[80] Bernstein A.M., Sun Q., Hu F.B., Stampfer M.J., Manson J.E., Willett W.C. (2010). Major dietary protein sources and risk of coronary heart disease in women. Circulation.

[81] Pan A., Sun Q., Bernstein A.M., Schulze M.B., Manson J.E., Stampfer M.J., Willett W.C., Hu F.B. (2012). Red meat consumption and mortality: Results from 2 prospective cohort studies. Arch. Intern. Med..

[82] Pan A., Sun Q., Bernstein A.M., Schulze M.B., Manson J.E., Willett W.C., Hu F.B. (2011). Red meat consumption and risk of type 2 diabetes: 3 cohorts of U.S. adults and an updated meta-analysis. Am. J. Clin. Nutr..

[83] Forouhi N.G., Harding A.H., Allison M., Sandhu M.S., Welch A., Luben R., Bingham S., Khaw K.T., Wareham N.J. (2007). Elevated serum ferritin levels predict new-onset type 2 diabetes: Results from the EPIC-Norfolk prospective study. Diabetologia.

[84] Shi Z., Yuan B., Zhang C., Zhou M., Holmboe-Ottesen G. (2011). Egg consumption and the risk of diabetes in adults, Jiangsu, China. Nutrition.

[85] Radzeviciene L., Ostrauskas R. (2012). Egg consumption and the risk of type 2 diabetes mellitus: A case-control study. Public Health Nutr..

[86] Expert Panel on Detection, Evaluation, and Treatment of High Blood Cholesterol in Adults (2001). Executive summary of the third report of the National Cholesterol Education Program (NCEP) Expert Panel on detection, evaluation, and treatment of high blood cholesterol in adults (Adult Treatment Panel III). JAMA.

[87] Grundy S., Becker D., Clark L.T., Cooper R.S., Denke M.A., Howard J., Hunninghake D.B., Illingworth D.R., Luepker R.V., McBride P. (2002). Third report of the National Cholesterol Education Program (NCEP) Expert Panel on detection, evaluation, and treatment of high blood cholesterol in adults (Adult Treatment Panel III) Final Report. Circulation.

[88] Krauss R.M., Deckelbaum R.J., Ernst N., Fisher E., Howard B.V., Knopp R.H., Kotchen T., Lichtenstein A.H., McGill H.C., Pearson T.A. (1996). Dietary guidelines for healthy American adults. A statement for health professionals from the Nutrition Committee, American heart association. Circulation.

[89] Luhovyy B.L., Akhavan T., Anderson G.H. (2007). Whey proteins in the regulation of food intake and satiety. J. Am. Coll. Nutr..

[90] Kuroda M., Sakaue H. (2016). Role of vitamin D and calciumin obesity and type 2 diabetes. Clin. Calcium.

[91] Berr C., Akbaraly T., Arnaud J., Hininger I., Roussel A.M., Gateau P.B. (2009). Increased selenium intake in elderly high fish consumers may account for health benefits previously ascribed to omega-3 fatty acids. J. Nutr. Health Aging.

[92] Bleys J., Navas-Acien A., Guallar E. (2007). Serum selenium and diabetes in U.S. adults. Diabetes Care.

[93] Bhathena S.J., Velasquez M.T. (2002). Beneficial role of dietary phytoestrogens in obesity and diabetes. Am. J. Clin. Nutr..

[94] Jayagopal V., Albertazzi P., Kilpatrick E.S., Howarth E.M., Jennings P.E., Hepburn D.A., Atkin S.L. (2002). Beneficial effects of soy phytoestrogen intake in postmenopausal women with type 2 diabetes. Diabetes Care.

