# Supplementary material for: Dietary Protein Consumption and the Risk of Type 2 Diabetes: A Systematic Review and Meta-Analysis of Cohort Studies
Source: Nutrients. 2017 Sep 6;9(9):982. doi: 10.3390/nu9090982 (PMC5622742; doi:10.3390/nu9090982)
Supplement: Supplementary file 1 [file nutrients-09-00982-s001.zip › nutrients-209795-supplementary.pdf]

Table S1. Characteristics of participants and follow-up in included studies of protein consumption in relation to risk of T2DM

Author	Publication year	Study name	country	Age at baseline (years)	No. of participant	No. of cases
Malik et al[15]	2016	Nurses' Health Study	USA	30-55	72992	7214
Malik et al[15]	2016	Nurses' Health Study Ⅱ	USA	24-42	92088	5032
Malik et al[15]	2016	Health Professionals Follow-up Study	USA	40-75	40722	3334
Akiko et al[17]	2015	Japan Public Health Center-Based Prospective Study	Jpapn	40-59	64674	1191
Monique et al [10]	2014	European Prospective Investigation into Cancer and Nurition and a stratified sub cohort	Europe		26253	11637
Ulrika et al [11]	2013	Malmo Diet and Cancer cohort	USA	47-74	27140	1709
Weo et al [12]	2013	Women's Health Study	USA	25-44	15294	870
Tinker et al [13]	2011	Women's Health Initiative	USA	50-79	74155	19111
Sluijs et al [14]	2010	European Prospective Investigation into Cancer and Nutrition(EPIC)-NL Study	Europe	21-64	38094	918

Table S2. Prospective cohort studies of protein consumption and type 2 diabetes.

study	Follow-up period	DM diagnosis and criteria	RR(95%CI) total	RR(95%CI) animal	RR(95%CI) plant	Adjustment variables
Malik et al, 2016(1)[15],USA	1984-2008,24y#	Self-report+ Supplementary questionnaires	1.04(0.96,1.13)	1.08(0.99,1.19)	0.91(0.52,1.02)	age,BMI,race,family history of diabetes, smoking status,alcohol intake, physical activity,total energy intake, menopausal status
Malik et al, 2016(2)[15],USA	1991-2009,18y#	Same as above	1.03(0.92,1.15)	1.11(0.98,1.25)	0.90(0.79,1.04)	
Malik et al, 2016(3)[15],USA	1986-2008,24y#	Same as above	1.18(1.04,1.34)	1.27(1.11,1.46)	0.91(0.77,1.07)	
Akiko et al, 2015[17] (men), Jpapn	1993-1998,5y 1993-2003,10y#	self-report+ examine medical records	1.36(0.97,2.14)	1.46(0.94,2.27)	0.88(0.30,1.30)	age,study area, BMI, smoking status,fat, carbohydrate, alcohol consumption,FH-DM total physical activity, history of hypertension
Akiko et al, 2015[16] (women),Japan	Same as above#	Same as above	0.87(0.55,1.38)	1.37(0.82,2.28)	0.91(0.61,1.35)	
Monique et al, 2014[10](men), Europe	1991-2007,12y § # &	Self-report	1.04(0.83,1.31)	1.01(0.71,1.43)	1.50(1.16,1.93)	age,energy,sex,smoking ,education,physical activity, alcohol, fiber, SFA,MUFA,PUFA,cholesterol, soft drink,tea,coffee,BMI,waist
Monique et al, 2014[10](women) Europe	Same as above § # &	Same as above	1.37(1.06,1.79)	1.38(1.01,1.90)	1.24(0.91,1.68)	

Table S2. (continued)						
**study**	**Follow-up period**	**DM diagnosis and criteria**	**RR(95%CI) total**	**RR(95%CI) animal**	**RR(95%CI) plant**	**Adjustment variables**
Ulrika et al, 2013[11](men), USA	12y#	regional Diabetes register of Scania	1.21(0.98,1.50)			age,method version, season,total energy, education,smoking,alcohol intake, leisure-time physical activity,BMI
Ulrika et al, 2013[11](women)	Same as above#	Same as above	1.30(1.03,1.64)			
Weo et al, 2013[12](women) USA	1991-2001,10y#	self- report	1.28(0.90,1.83)	1.49(1.03,2.17)	0.69(0.50,0.97)	age,parity,race,family history of diabetes,cigarette smoking alcohol intake,physical activity,total energy intake, saturate fat,monounsaturated fat,poluunsaturate fat
Tinker et al, 2011[13](women) USA	1993-1995,5y#	Report at enrollment	1.16(1.05,1.28)			physical activity, race-ethnicity, education, income, history of cardiovascular disease, smoking status,
Sluijs et al, 2010[14], Europe	1993-1997,5y#	self-report+hospital diagnoses	1.16(1.02,1.76)	1.13(1.04,1.22)	1.04(0.83,1.29)	age, sex,dietary intake,waist ,BMI, energy intake,saturated fat, monounsaturated fat, polyunsaturated fat, cholesterol, vitamin E

FH-DM, family history of diabetes; W, women; M, men; SFA, saturated fatty acid; MUFA, monounsaturated fatty acid; PUFA, polyunsaturated fatty acid
#Diet assessment method was FFQ. §Diet assessment method was 7-day menu book. &Diet assessment method was 45-min interview.

Table S3. Characteristics of participants and follow-up in included studies of meat consumption in relation to risk of T2DM

Author	Publication year	Study name	country	Age at baseline (years)	No. of participant	No. of cases
Van Dan et al [19]	2012	Health Professionals' Follow-up stud	USA	40-75	42501	1321
Schulze et al [22]	2003	Nurses' Health Study Ⅱ	USA	26-46	91246	741
Lee et al [26]	2004	Women's Health Study	USA	55-69	35698	1921
Fung et al [21]	2004	Nurses' Health Study	USA	55-69	35698	1921
Song et al [23]	2004	Women's Health Study	USA	≥45	37309	1558
Montonen et al[28]	2005	Finnish Mobile Clinic Health Examination Survey	Finland	40-69	4304	382
Villegas et al [24]	2006	Shanghai Women's Health Study	China	40-70	70909	1972
Hodge et al [20]	2007	Melbourne Collaborative Cohort Study	Australia	27-75	31641	365
Schulze et al [27]	2007	EPIC-Potsdam Cohort Study	Germany	35-65	25167	849
Simmons et al [25]	2007	EPIC-Norfolk Cohort Study	Uk	40-79	25038	417
Vang et al [29]	2008	Adventist Mortality Study, Adventist Health Study	USA	45-88	8701	543

Table S4. Prospective cohort studies of meat consumption and type 2 diabetes					
**study**	**Follow-up period**	**DM diagnosis and criteria**	**RR(95%CI) Red meat**	**RR(95%CI) Processed meat**	**Adjustment variables**
Van Dan, et al, 2012[19], USA	1986-1998, 12 y#	self-report+Supplemental questionnaires WHO 1985, validated DM2 status	1.04(0.96,1.13)	1.05(0.85-1.30)	Age, total energy, time period, physical activity, cigarette smoking, alcohol, hypercholesterolaemia, cereal hypertension, FH-DM2, fiber, magnesium, BMI
Schulze, et al, 2003[22], USA	1991-1999, 8y#	Self-report+supplemental questionnaires,validated method for assessment of DMS NDDG	1.82(1.34,2.46)	1.44(0.92,2.24)	Age, BMI, FH-DM, alcohol, smoking, menopausal status, dietary energy, hypertension, physical activity, HRT, OC use.cereal fiber, magnesium, caffeine, glycaemic index, hypercholesterolaemia,
Lee, et al, 2004[26], USA	1986-1997. 11y#	Self-report	1.19(0.97-1.45)		Age, total energy, WHR, BMI, physical activity,HRT, cigarette smoking, alcohol, education, marital status, residential area, animal fat, vegetable fat, cereal fiber, magnesium
Fung, et al, 2004[21], USA	1984-1998, 14y#	self-report+supplemental questionnaires NDDG, validated DM2 status	1.36(1.18,1.56)	1.60(1.39,1.83)	Age, FH-DM2, smoking, menopausal status, dietary, hypercholesterolaemia, hypertension, physical activity, alcohol, BMI, missing FFQ
Song, et al, 2004[23] , USA	1993-2003, 8y#	Self-report+ supplemental questionnaires.Blood samples in subgroup using ADA, validated DMS status	1.25(0.94,1.67)	1.38(1.11,1.71)	age,study area, BMI, smoking status,fat, carbohydrate, alcohol consumption,FH-DM total physical activity, history of hypertension

Table S4. (continued)					
**study**	**Follow-up period**	**DM diagnosis and criteria**	**RR(95%CI) Red meat**	**RR(95%CI) Processed meat**	**Adjustment variables**
Montonen, et al, 2005[28] , Finland	1967-1990, 23 y#	Identified from register of persons receiving drug imbursement	1.22(0.89,1.69)	0.99(0.72,1.38)	Age, sex, BMI, energy intake, smoking, FH-DM, geographic area
Villegas, et al, 2006[24], China	1997-2000-2004, 4.6y, 326581 person-years #	Self-report analysis of 1094 cases confirmed by ADA criteria yielded similar results to all cases	0.94(0.79,1.12)	1.18(0.99,1.37)	Age, energy, BMI, WHR, smoking, alcohol, physical activity, vegetable intake, income level, education level, occupation status, hypertension
Hodge, et al, 2007[20],Australia	1990-1994, 4y#	Self-report+physician confirmation for 76% of cases	0.99(0.72,1.38)	1.22(0.89,1.69)	Age, energy intake, FH-DM, country of birth, BMI, WHR
Schulze, et al, 2007[27], Germany	1994-1998-2005,7y #	Self-report+physician verification	1.64(1.23,2.19)		Age, height, alcohol, coffee, waist circumference, sports, hypertension, whole grains, biking, gardening, smoking
Simmons, et al, 2007[25],Uk	1993-1998, 4.6y, 115137 person-years #	Self-report, health checks, hospital and general practice registers, HbA1c>7%, medications	2.26(1.10,4.66)	2.95(1.39,6.25)	Crude

Table S4. (continued)					
**study**	**Follow-up period**	**DM diagnosis and criteria**	**RR(95%CI) Red meat**	**RR(95%CI) Processed meat**	**Adjustment variables**
Vang, et al, 2008[29], USA	1960-1976, 17 y#	Self-report	1.27(1.06,1.53)	1.38(1.05,1.82)	Age, sex, Exclusion of smokers, alcohol users and those with pre-existing disease did not alter the results

FH-DM, family history of diabetes; FH-DM2, family history of type 2 diabetes; M, men; W, women; NA, not available; HRT, hormone replacement therapy; OC use, oral contraceptive use

#Diet assessment method was FFQ

Table S5. Characteristics of participants and follow-up in included studies of fish consumption in relation to risk of T2DM

Author	Publication year	Study name	country	Age at baseline (years)	No. of participant	No. of cases
Montonen et al[28]	2005	Finnish Mobile Clinic Health Examination Survey	Finland	40-69	4301	383
Kaushik et al[36]	2009	Nurses' Health Study	USA	30-55	61031	4159
Kaushik et al[36]	2009	Nurses' Health Study II	USA	26-46	91669	2728
Kaushik et al[36]	2009	Helath Professionals Follow-up Study	USA	39-78	42504	2493
Patel et al [35]	2009	EPIC-Norfolk	England	40-79	21442	700
Geertruida et al[34]	2005	The Netherlands, Rotterdam Study	Netherlands	≥55	4472	463
Djousse et al [31]	2011	Women's Health Study	USA	≥ 45	36328	2370
Villegas et al [32]	2011	Shanghai Women's Health Study	China	40-70	64193	3034
Villegas et al [32]	2011	Shanghai men's Health Study	China	40-74	51963	900
Nanri et al [33]	2011	Public Health Center-based Prospective Study	Japan	45-75	52680	971
Wallin et al [30]	2015	The Cohort of Swedish Men	Swedish	45-79	35583	3624

Table S6. Prospective cohort studies of fish consumption and type 2 diabetes

study	Follow-up period	DM diagnosis and criteria	RR(95%CI)	Adjustment variables
Montonen, et al, 2005[28],Finland	1967-1995, 28 y#	Fasting glucose level ≥7.0mmol/L, plasma glucose level ≥11.1 mmol/L, treatment by diet	1.08(0.80,1.45)	Age, sex, BMI, total energy, smoking, FH-DM, geographic area(hypertension, serum cholesterol, self-rated health occupation
Kaushik, et al, 2009(1)[36],USA	1986-2005, 20y#	Self report+ Fasting glucose level≥7.0mmol/L, plasma glucose level ≥11.1 mmol/L	1.29(1.05,1.57)	Age, BMI, physical activity, saturated fat, trans fat, linolenic acid, caffeine, cereal fiber, glycemic index, alcohol, total energy, smoking,FH-DM, hormone replacement therapy, waist circumference, ratio of polyunsaturated to saturated fat, processed meat, carbonated soft drink
Kaushik, et al, 2009(2)[36],USA	1986-2005, 20y#	Same as above	1.32(0.99,1.74)	
Kaushik, et al, 2009(3)[36],USA	1986-2005, 20y#	Same as above	1.16(0.96,1.41)	
Patel, et al, 2009[35] , England	Same as above#	Self-report+diagnosis captured	0.75(0.58,0.96)	Age, sex, BMI, waist circumference, physical activity, plasma vitamin C, alcohol, total energy, smoking, family history of diabetes, education
Geertruida,et al, 2009[34] Netherlands	1990-2005, 15y #	Fasting glucose leve l≥7.0mmol/L, plasma glucose level ≥11.1 mmol/L, treatment by diet,diabetes register	1.05(0.80,1.38)	Age, sex, trans fat, fiber, alcohol, total energy, smoking, education, BMI, waist circumference, saturated fat, α-linolenic acid, linoleic acid, fruit, vegetable, meat, coffee, medically prescribed diet, hypertension, total cholesterol, HDL-C, FH-DM

Table S6. (continued)				
**study**	**Follow-up period**	**DM diagnosis and criteria**	**RR(95%CI)**	**Adjustment variables**
Djousse , et al, 2011[31](men), USA	1992-2008, 16y #	Fasting plasma glucose ≥7.8mmol/L, casual plasma glucose≥11mmol/L	0.73(0.54,1.00)	Age, sex, BMI, physical activity, alcohol, total energy, smoking, LDL-C, clinic site, race
Djousse , et al, 2011[31](women), USA	Same as above#	Same as above	1.01(0.69,1.49)	
Nanri, et al 2011[33], (men) Japan	1995-2003,8y#	Fasting plasma glucose≥7.8mmol/L, casual plasma glucose≥11mmol/L	0.73(0.54,1.00)	Age, sex, BMI, physical activity, alcohol, total energy, smoking, LDL-C, clinic site, race
Nanri, et al 2011[33], (men) Japan	Same as above#	Same as above	1.01(0.69,1.49)	
Villegas, et al, 2011[32](women), China	1996-2006, 10y#	Fasting glucose concentration≥7mmol/L on ≥2 separate occasions, oral-glucose-tolerance test ≥ 11.1 mmol/L, use of hypoglycemic medication	0.86(0.76,0.98)	Age,sex, BMI, waist circumference, physical activity, plasma vitamin C, alcohol, total energy, smoking, FH-DM, education
Villegas, et al, 2011[32](men), China	2002-2008, 6y#	Same as above	0.92(0.73,1.16)	

**Table S6. (continued)**				
**study**	**Follow-up period**	**DM diagnosis and criteria**	**RR(95%CI)**	**Adjustment variables**
Wallin, et al, 2015[30](men) Swedish	1998-2012,15y#	Diabetes register	1.00(0.85,1.18)	Age, BMI, physical activity, education, cigarette smoking, total energy intake, intake of alcohol, DASH diet component score(based on intake of fruits, vegetables, nuts, legumes, low-fat dairy, whole grains, sodium, sweetened beverages, red and processed meat)

FH-DM, family history of diabetes; W, women; M, men; SFA, saturated fatty acid; MUFA, monounsaturated fatty acid; PUFA, polyunsaturated fatty acid

#Diet assessment method was FFQ.

Table S7. Characteristics of participants and follow-up in included studies of egg consumption in relation to risk of T2DM

Author	Publication year	Study name	country	Age at baseline (years)	No. of participant	No. of cases
Djousse et al [37]	2009	Physicians' Health Study I	USA	>40	20703	1921
Djousse et al [37]	2009	Women's Health Study	USA	>45	36295	2112
Djousse et al [38]	2010	Cardiovascular Health Study	USA	>65	3898	303
Djousse et al[39]	2016	The Jackson Heart Study	USA	21-95	4568	513
Virtanen et al[40]	2015	Kuopio Ischaemic Heart Disease Risk Factor Study	Finland	42-60	2332	432

Table S8. Prospective cohort studies of egg consumption and type 2 diabetes				
**study**	**Follow-up period**	**DM diagnosis and criteria**	**RR(95%CI)**	**Adjustment variables**
Djousse, et al, 2009(1)[37](men), USA	1982-2007, mean 20y#	Self-report or medical record	1.58(1.25,2.01)	Age, smoking, alcohol, physical activity, BMI, FH-DM, hyperliidemia, hypertension, red meat intake, energy, fruits and vegetables, saturated fatty acids, trans fatty acids, polyunsaturated fatty acids
Djousse, et al, 2009(2)[37] (women), USA	1992-2007, mean 11.7y#	Same as above	1.77(1.28,2.43)	
Djousse, et al, 2010(1)[38](men), USA	1989-2007, mean 11.3y#	Medical record	1.81(0.77,4.22)	Age, race, field center, BMI, physical activity, energy, smoking alcohol, fiber intake
Djousse, et al, 2010(2)[38] (women), USA	Same as above#	Same as above	0.38(0.10,1.37)	
Djousse, et al, 2016[39] ,USA	2000-2004#	Fasting glucose≥126mg/dL,hemoglobin A1C≥6.5%,or use of insulin or oral hypoglycemic	1.52(1.17,1.97)	Age, smoking, alcohol, BMI, physical activity score, education, energy intake, intake of red meat and fruits and vegetables, dietary trans fat, magnesium, fiber, history of hypertension, CVD

Table S8. (continued)

study	Follow-up period	DM diagnosis and criteria	RR(95%CI)	Adjustment variables
Virtanen, et al, 2015[40],Finland	1984-1989, 19.3y #	Self-report+fasting plasma glucose≥7.0mmol/L or 2-h oral-glucose-tolerance-test plasma glucose≥11.1mmol/L	0.38(0.27,0.53)	Age, examination year, energy intake, BMI, FH-DM2, hypertension, smoking, education year, leisure-time physical activity, serum long-chain omega-3, PUFAs, intake of alcohol, linoleic acid, fiber,fruit, berries, vegetables, dietary cholesterol intake.

FH-DM, family history of diabetes; FH-DM2, family history of type 2 diabetes; M, men; W, women; CVD,cardiovascular disease; PUFA, polyunsaturated fatty acid; CVD,cardiovascular disease;

#Diet assessment method was FFQ.

Table S9. Characteristics of participants and follow-up in included studies of dairy consumption in relation to risk of T2DM						
**Author**	**Publication year**	**Study name**	**country**	**Age at baseline (years)**	**No. of participant**	**No. of cases**
Grantham et al[41]	2013	the Australian Diabetes Obesity and Lifestyle Study	Australia	≥25	5582	209
Choi et al [42]	2005	Health Professionals Follow-up Study	USA	mean age 53	41254	1234
Liu et al [43]	2006	Women's Health Study	USA	mean age 55	37183	1603
Van Dam et al [44]	2006	Black Women's Health Study	USA	mean age 3	41186	1964
Elwood et al [48]	2007	Caerphilly prospective study	UK	45-59	640	41
Kirii et al [47]	2009	Japan Public Health Center-based Prospective Study	Japan	mean age 57	25877	634
Kirii et al [47]	2016	Japan Public Health Center-based Prospective Study	Japan	mean age 57	33919	480
Dial-Lopez et al [45]	2011	Shanghai men's Health Study	Spanish	55-80	3454	270
Karen et al [46]	2011	Women's Health Study	USA	40-79	93676	3946

Table S10. Prospective cohort studies of dairy consumption and type 2 diabetes

study	Follow-up period	DM diagnosis and criteria	RR(95%CI) Total dairy product	RR(95%CI) Whole milk	RR(95%CI) yogurt	Adjustment variables
Grantham, et al, 2013(1)[41](men) Australia	1999-2000-2005, 5y#	Fasting plasma glucose ≥7.0mmol/L or 2h post-load plasma glucose ≥11.1mmol/L or current treatment with insulin or oral hypoglycaemic agents	0.53(0.29,0.96)	1.43(0.83,2.45) 0.57(0.32,1.02)	1.02(0.56,1.88)	Age ,sex, total energy intake, FH-DM, education, physical activity, smoking status, fasting serum TAG, HDL-C, systolic blood pressure, waist circumference, hip circumference
Grantham, et al, 2013(2)[41](women) ,Australia	Same as above#	Same as above	0.86(0.50,1.45)	0.89(0.45,1.75) 0.67(0.40,1.12)	1.23(0.74,2.04)	
Choi, et al, 2005[42](men), USA	12y#	Fasting plasma glucose ≥7.8mmol/l and/or 2h post-load plasma glucose ≥11.1mmol/l or treatment with insulin or oral hypoglycemic medication	0.77(0.62,0.95)	0.97(0.78,1.21) 0.73(0.59,0.89)	0.83(0.66,1.06)	Age, energy, biennial follow-up time, FH-DM, smoking, BMI, hypertension, physical activity, alcohol, dietary factors, hypercholesterolemia
Liu, et al, 2006[43] (men), USA	10y#	Fasting plasma glucose≥7.0mmol/L or 2h post-load plasma glucose ≥11.1mmol/L	0.79(0.64,0.94)	1.05(0.88,1.24) 0.79(0.67,0.93)	0.82(0.70,0.97)	Age, energy, BMI, smoking randomized-treatment assignment, FH-DM, hypercholesterolemia, hypertension, physical activity, hormones, alcohol consumption

Table S10. (continued)

study	Follow-up period	DM diagnosis and criteria	RR(95%CI) Total dairy product	RR(95%CI) Whole milk	RR(95%CI) yogurt	Adjustment variables
Van Dam, et al, 2006[44] (women),USA	8y#	Fasting plasma glucose ≥7.0mmol/L or 2h post-load plasma glucose ≥11.1mmol/L	0.93(0.75,1.15)	1.03(0.88,1.20)<br>0.87(0.76,1.00)		Age, energy, BMI, smoking, physical activity, alcohol consumption, parental history of diabetes, education, coffee, diet.
Elwood, et al, 2007[48](men), UK	25y #	Plasma insulin level ≥163mmol/l and/or plasma glucose ≥6.1mmol/l	0.57(0.20,1.63)			Age, smoking, BMI, social class
Kirii, et al, 2009[47](men), Japan	12y#	Fasting plasma glucose≥7.8mmol/l and casual plasma glucose≥11mmol/l	1.18(0.90,1.56)		1.02(0.85,1.24)	Age, area, BMI, FH-DM, smoking, alcohol, history of hypertension, exercise frequency, coffee, energy-adjusted magnesium and total energy
Kirii, et al, 2009[47](women), Japan	Same as above#	Same as above	0.71(0.51,0.98)		0.87(0.70,1.09)	

Table S10. (continued)

study	Follow-up period	DM diagnosis and criteria	RR(95%CI) Total dairy product	RR(95%CI) Whole milk	RR(95%CI) yogurt	Adjustment variables
Dial-Lopez, et al, 2016[45] Spanish	4.1y#	Fasting plasma glucose≥7.0mmol/L or 2h post-load plasma glucose ≥11.1mmol/L	0.68(0.47,0.98)	0.65(0.45,0.94)	0.60(0.42,0.86)	Age, race/ethnicity, total energy intake, income, education, smoking, alcohol consumption, use of postmenopausal hormone, physical activity, FH-DM, BMI, blood pressure.
Karen, et al, 2011[46](women) USA	1993-1995,5y#	Self- report+ treated with insulin or oral medication for diabetes	0.93(0.83,1.04)	0.65(0.44,0.96)	0.46(0.31,0.68)	Age, race/ethnicity, total energy intake, income, education, smoking, alcohol consumption, use of postmenopausal hormone, physical activity, FH-DM, BMI, blood pressure.

FH-DM, family history of diabetes; W, women; M, men;HDL-C, high density lipoprotein cholesterol
#Diet assessment method was FFQ.

Table S11. Characteristics of participants and follow-up in included studies of soy consumption in relation to risk of T2DM

Author	Publication year	Study name	country	Age at baseline (years)	No. of participant	No. of cases
Stuapa et al [50]	2013	India's third National Family Health Survey	India	20-49	99574	56742
Villegas et al [51]	2008	Health Professionals Follow-up Study	China	middle-aged	64227	1608
Mueller et al [52]	2012	The Singapore Chinese Health Study(SCHS)	Singapore	mean age 55.2	43176	2252
M aldwairji et al [53]	2013	The UK Women Cohort's Study	UK	middle age	12149	
Morimoto et al [53]	2011	the Multiethnic Cohort.	USA	45-75	29719 Caucasian	8594
Morimoto et al [53]	2011	the Multiethnic Cohort.	USA	45-75	35141 Japanese	
Morimoto et al [53]	2011	the Multiethnic Cohort.	USA	45-75	10484 Native	

Table S12. Prospective cohort studies of soy consumption and type 2 diabetes

study	Follow-up period	DM diagnosis and criteria	RR(95%CI)	Adjustment variables
Stuapa, et al, 2013(1)[49](men) India	1y#	Self report	0.70(0.39,1.26)	BMI, tobacco smoking, alcohol drinking, age, watching TV, education, living standard of the household, residence, consumption of other food items.
Stuapa, et al, 2013(2)[49] (women) India	Same as above#	Same as above	0.55(0.34,0.88)	
Villegas, et al, 2008[50](women), China	2000-2003- 2004 Average 4.6y#	Fasting glucose concentration≥ 7mmol/L, oral-glucose-tolerance test≥11.1mmol/L, use of hypoglycemic medication	0.62(0.51, 0.75)	Age, race, field center, BMI, physical activity, energy, smoking alcohol, fiber intake
Mueller, et al, 2012[51], Singapore	1999-2004, 6y#	Self-report	0.86(0.67,1.10)	Age, sex, dialect, year of interview, education level, smoking status, alcohol use, physical activity, baseline hypertensive, rice, noodles, vegetable, energy
M aldwairji, et al,2013[52] (women),UK	Average 4 y	Self-report	0.49(0.29,0.84)	Energy, smoking status, adult weight change, socioeconomic status, alcohol intake, physical activity, FH-DM
Morimoto, et al, 2011[53](men), USA	1993-1996-2009, 14y #	Self- report+ treated with insulin or oral medication for diabetes	1.18(1.08,1.29)	

Table S12. (continued)

study	Follow-up period	DM diagnosis and criteria	RR(95%CI)	Adjustment variables
Morimoto, et al, 2011[53](women), USA	Same as above #	Same as above	1.18(1.08,1.29)	

FH-DM, family history of diabetes; DM, diabetes; W, women; M, men
#Diet assessment method was FFQ.
